# Using Baidu search values to monitor and predict the confirmed cases of COVID-19 in China: – evidence from Baidu index

**DOI:** 10.1186/s12879-020-05740-x

**Published:** 2021-01-21

**Authors:** Bizhi Tu, Laifu Wei, Yaya Jia, Jun Qian

**Affiliations:** 1 Department of Orthopedics, The First Affiliated Hospital of Anhui Medical University, 218 Jixi Road, Hefei, 230022 Anhui China; 2 Department of Pediatrics, The Shanxi Medical University, Taiyuan, Shanxi China

**Keywords:** COVID-19, Web-based data, Internet searching, Baidu index

## Abstract

**Background:**

New coronavirus disease 2019 (COVID-19) has posed a severe threat to human life and caused a global pandemic. The current research aimed to explore whether the search-engine query patterns could serve as a potential tool for monitoring the outbreak of COVID-19.

**Methods:**

We collected the number of COVID-19 confirmed cases between January 11, 2020, and April 22, 2020, from the Center for Systems Science and Engineering (CSSE) at Johns Hopkins University (JHU). The search index values of the most common symptoms of COVID-19 (e.g., fever, cough, fatigue) were retrieved from the Baidu Index. Spearman’s correlation analysis was used to analyze the association between the Baidu index values for each COVID-19-related symptom and the number of confirmed cases. Regional distributions among 34 provinces/ regions in China were also analyzed.

**Results:**

Daily growth of confirmed cases and Baidu index values for each COVID-19-related symptom presented robust positive correlations during the outbreak (fever: *r*_s_=0.705, *p*=9.623× 10^− 6^; cough: *r*_s_=0.592, *p*=4.485× 10^− 4^; fatigue: *r*_s_=0.629, *p*=1.494× 10^− 4^; sputum production: *r*_s_=0.648, *p*=8.206× 10^− 5^; shortness of breath: *r*_s_=0.656, *p*=6.182× 10^–5^). The average search-to-confirmed interval (STCI) was 19.8 days in China. The daily Baidu Index value’s optimal time lags were the 4 days for cough, 2 days for fatigue, 3 days for sputum production, 1 day for shortness of breath, and 0 days for fever.

**Conclusion:**

The searches of COVID-19-related symptoms on the Baidu search engine were significantly correlated to the number of confirmed cases. Since the Baidu search engine could reflect the public’s attention to the pandemic and the regional epidemics of viruses, relevant departments need to pay more attention to areas with high searches of COVID-19-related symptoms and take precautionary measures to prevent these potentially infected persons from further spreading.

**Supplementary Information:**

The online version contains supplementary material available at 10.1186/s12879-020-05740-x.

## Background

The outbreak of new coronavirus disease 2019 (COVID-19) was characterized by fever, cough, fatigue, sputum production, and shortness of breath, receiving people’s attention globally [1, 2]. As of April 22, 2020, COVID-19 had spread to more than 188 countries and regions, resulting in over 9.6 million confirmed cases and 490 thousand deaths worldwide [3]. The astonishing spread speed of the epidemic, to some extent, was failing to monitor and manage the potentially infected persons, which may pose a substantial infection control challenge [4]. Therefore, recognizing the potential quantity of infected persons timely and taking corresponding management measures to control the further spread of COVID-19 is in urgent need.

Because of the unpredictability of international public health emergency, novel methods for monitoring the epidemic’s development are substantial. Network real-time data can be easily obtained from the web due to the quick availability of the Internet. According to the 45th China statistical report on internet development, there were over 904 million Internet users in China, with the penetration rate of search engine use reached 83% [5]. Among Internet users, 80% of them tended to use electronic devices to acquire the information they are interested in [6].

Recently, people can easily get health-related information via Internet search engines, which could greatly reflect the searches’ physical condition or the relatives and friends the searchers concerned [7]. Moreover, to interrupt the transmission of the epidemic, the Chinese government had put in place strong quarantine measures, which also influences the routinely outpatient service process. Reported studies showed that public search behaviors have already been used to predict some epidemic diseases, such as influenza [8], epidemic erythromelalgia [9], dengue [10], and HIV/AIDS [11].

The surveillance of network searches about clinical symptoms of COVID-19 is more predictable and timely compared to previous detection surveillance (e.g., official announcements, news reports, and mass media) [12–14]. Baidu serves as the most popular search engine, occupies more than 90% of Internet users in China [15]. In this study, we obtained the Baidu index values of COVID-19-related symptoms and the data of confirmed cases of COVID-19 across China to analyze the association between these variables and explore whether the Baidu index could act as a novel tool for monitoring and predicting the epidemic of COVID-19 in China.

## Methods

### Data from Baidu index

More than 90% of Chinese search engine users tend to use Baidu to retrieve their interesting information [16, 17]. The weighted sum of the Baidu search values can describe the characteristics of people’s search behaviors [18]. Baidu Index value is obtained by calculating the number of searches of specific keywords input by the searchers [18]. Using the keywords analysis function, Baidu Index automatically matches its related words according to the keywords typed by users. Previous studies had reported that the top five most common symptoms of the COVID-19 were fever (which accounted for 88.7% of the confirmed cases during hospitalization), cough (67.8%), fatigue (38.1%), sputum production (33.7%), and shortness of breath (18.7%) [1]. Accordingly, we selected those symptoms as the keywords in the current study. Based on the keyword analysis function, 26 search terms that could represent the most common symptoms of COVID-19 were selected (Table S1). We added the search values of each symptom and its related keywords together to get the composite Baidu Index values to perform our research. Besides, we compared the search values of 5 keywords between 2011 and 2020 vertically to investigate whether the Baidu Index changes were an accidental event during the outbreak (Figure S1). To explore whether people’s search behaviors appear earlier than the epidemic of COVID-19, we defined a definition to examine our hypothesis: search-to-confirmed interval (STCI). The values of STCI can be obtained by calculating the time interval between the peak growth rate (daily Baidu Index values (DBIV) minus its previous day’s values as the growth rate) of the Baidu Index and the peak daily growth of confirmed cases (DGCC). The top ten provinces/regions ranked by the cumulative confirmed cases were selected for STCI analysis.

### Confirmed cases of COVID-19

We obtained the data of confirmed cases of COVID-19 from accessible official channels, including the official website of Hopkins University [2], the world health organization (WHO) [19], and the National Health Commission of the People’s Republic of China [20]. Since China’s epidemic had been gradually controlled after April 22, 2020, we divided the COVID-19 pandemic (January 11, 2020, to April 22, 2020) into a growth period and a decline period. February 10, 2020, was set as the cut-off date, when the government announced the road closures re-opened and fully production resumed [21].

### Statistical analysis

Using SPSS (version 23.0), we applied a Spearman correlation analysis to explore the relationships between DGCC and DBIV of COVID-19-related symptoms from January 11, 2020, to April 22, 2020. Using the same statistical methods, we also explored the time lag pattern between DGCC and DBIV of COVID-19-related symptoms. *P*< 0.05 was set as the level of statistical significance (two-sided test). Besides, GraphPad Prism 8.2 was used to draw figures.

## Results

### Correlation analysis among search values of Baidu index, cumulative confirmed cases and DGCC in China

**Fig. 1:**
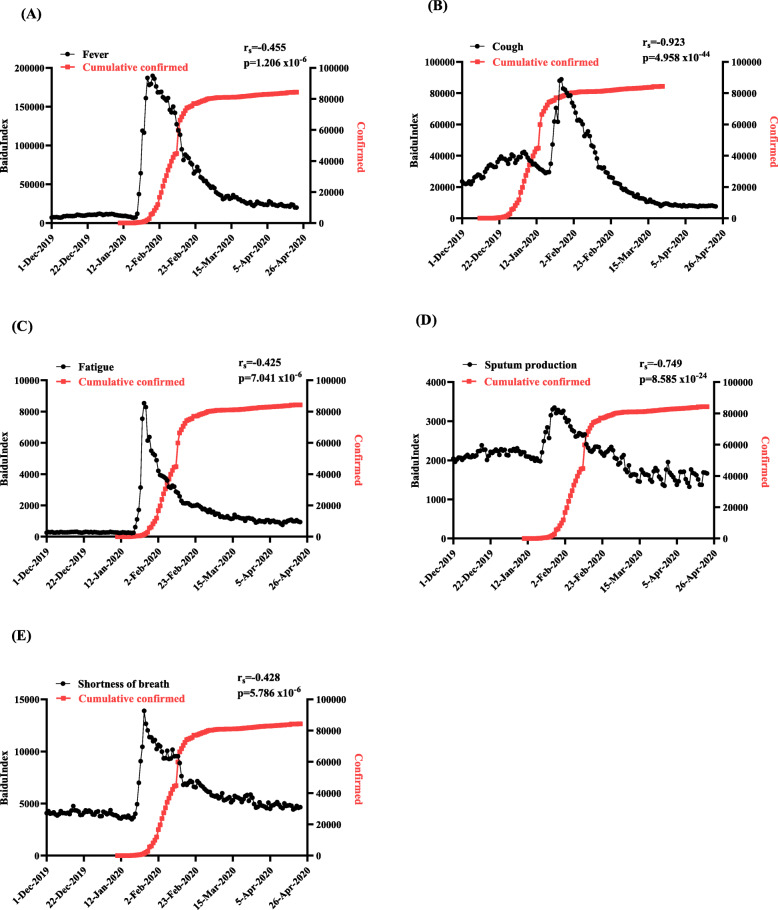
Correlation and time plots among cumulative confirmed cases and each keyword of COVID-19-related symptoms. **a**-**e** represent Baidu searches for “fever”, “cough”, “fatigue”, “sputum production”, and “shortness of breath”, repectively

As shown in Fig. 1, nationwide cumulative confirmed cases were strongly negative correlated to DBIV (fever: *r*_*s*_=− 0.455, *p*=1.206× 10^− 6^; cough: *r*_*s*_=− 0.923, *p*=4.958× 10^− 44^; fatigue: *r*_*s*_=− 0.425 *p*=7.041× 10^− 6^; sputum production: *r*_*s*_=− 0.749, *p*=8.585× 10^− 24^; shortness of breath: *r*_*s*_*=*-0.428, *p=*5.786× 10^− 6^). Taking the cut-off date (February 10, 2020) as the demarcation point, the cumulative confirmed cases and DBIV of fever (*r*_*s*_=0.705, *p*=9.623× 10^− 6^), cough (*r*_*s*_=0.592, *p*=4.485× 10^− 4^), fatigue (*r*_*s*_*=*0.629 *p=*1.494× 10^− 4^), sputum production (*r*_*s*_=0.648, *p=*8.206× 10^− 5^), shortness of breath (*r*_*s*_=0.656, *p=*6.182× 10^− 5^) had a strong positive correlation during the growth period and a significantly negative correlation during the decline period (fever: *r*_*s*_=− 0.971, *p* =5.850× 10^− 46^; cough: *r*_*s*_=− 0.967, *p*=8.601× 10^− 44^; fatigue: *r*_*s*_=− 0.937 *p* =3.948× 10^− 34^; sputum production: *r*_*s*_=− 0.770, *p*=1.604 × 10^− 15^; shortness of breath: *r*_*s*_=-0.930, *p=*1.333× 10^− 32^) (Figures S2 and S3).

**Fig. 2:**
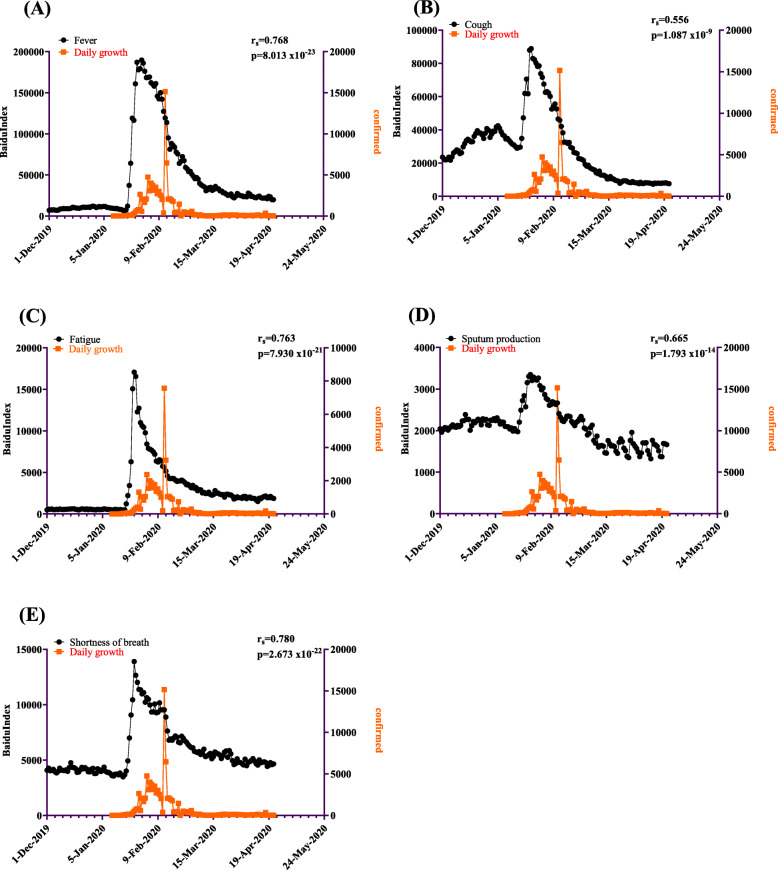
Correlation and time plots among daily confirmed cases and each keyword of COVID-19-related symptoms. **a**-**e** represent Baidu searches for “fever”, “cough”, “fatigue”,“sputum production”, and “shortness of breath”, repectively

Table 1 and Fig. 2 shows that there were strong statistically positive correlations among the DGCC and Baidu search values of fever (*r*_*s*_=0.768, *p=*8.013× 10^− 23^), cough (*r*_*s*_=0.556, *p=*1.087× 10^− 9^), fatigue (*r*_*s*_=0.763, *p=*7.930× 10^− 21^), sputum production (*r*_*s*_=0.665, *p*=1.793× 10^− 14^), and shortness of breath (*r*_*s*_=0.780, *p*=2.673× 10^− 22^), nationwide. Among the 34 provinces/regions in China, we found significant correlations between DGCC and DBIV; we observed that the number of daily confirmed cases tended to increase when Baidu searches for terms related to fever, cough, fatigue, and shortness of breath were increasing (Table 1). For Hong Kong, Macao, Taiwan, and Tibet, no consistent correlation was detected between DGCC and Baidu search values of COVID-19-related symptoms. However, DBIV of cough in Shanghai was not correlated to DGCC (*r*_*s*_=0.133, *p=*0.184). Besides, the correlations between sputum production and DGCC in several provinces/regions were inconspicuous (e.g., Beijing: *r*_*s*_=0.249, *p=*0.012; Guangdong: *r*_s_=0.262, *p=*0.008, Hunan: *r*_*s*_=-0.244, *p=*0.014) (Table 1).

**Table 1:** Correlation between daily growth of confirmed cases (DGCC) across China and values of Baidu index (BI)

Region	Values of BI				
	Fever	Cough	Fatigue	Sputum production	Shortness of breath
**China**					
*r*_*s*_	0.768	0.556	0.763	0.665	0.780
*p*	8.013 × 10^− 23^	1.087 × 10^− 9^	7.930 × 10^− 21^	1.793 × 10^− 14^	2.673 × 10^−22^
**Anhui**					
*r*_*s*_	0.801	0.770	0.760	− 0.028	0.775
*p*	5.39 × 10^24^	3.131 × 10^− 21^	2.172 × 10^− 20^	0.782	1.205 × 10^− 21^
**Beijing**					
*r*_*s*_	0.657	0.431	0.582	0.249	0.610
*p*	6.336 × 10^−14^	6.502 × 10^− 6^	1.358 × 10^−10^	0.012	1.040 × 10^− 11^
**Chongqing**					
*r*_*s*_	0.796	0.769	0.740	0.572	0.738
*p*	1.542 × 10^−23^	1.647 × 10^− 23^	6.057 × 10^− 19^	3.389 × 10^− 10^	8.809 × 10^− 19^
**Fujian**					
*r*_*s*_	0.588	0.471	0.705	0.367	0.537
*p*	8.473 × 10^− 11^	5.677 × 10^− 7^	1.388 × 10^− 16^	1.485 × 10^− 4^	5.809 × 10^− 9^
**Gansu**					
*r*_*s*_	0.527	0.444	0.373	− 0.150	0.484
*p*	1.277 × 10^− 8^	3.008 × 10^− 6^	1.112 × 10^− 4^	0.133	2.586 × 10^− 7^
**Guangdong**					
*r*_*s*_	0.535	0.336	0.527	0.262	0.506
*p*	7.113 × 10^−9^	1.564 × 10^− 4^	1.287 × 10^− 8^	0.008	5.598 × 10^− 8^
**Guangxi**					
*r*_*s*_	0.766	0.754	0.760	0.287	0.731
*p*	7.075 × 10^−21^	5.780 × 10^−20^	1.904 × 10^− 20^	0.004	6.872 × 10^− 8^
**Guizhou**					
*r*_*s*_	0.673	0.657	0.622	0.355	0.629
*p*	9.182 × 10^− 15^	6.433 × 10^− 14^	2.921 × 10^− 12^	2.555 × 10^− 4^	1.388 × 10^− 12^
**Hainan**					
*r*_*s*_	0.717	0.735	0.694	−0.354	0.693
*p*	2.474 × 10^− 17^	1.468 × 10^− 18^	6.080 × 10^− 16^	2.673 × 10^− 4^	6.597 × 10^− 16^
**Hebei**					
*r*_*s*_	0.731	0.635	0.662	0.040	0.705
*p*	2.622 × 10^−18^	7.392 × 10^− 13^	3.396 × 10^− 14^	0.691	1.297 × 10^− 16^
**Heilongjiang**					
*r*_*s*_	0.413	0.201	0.453	0.089	0.345
*p*	1.590 × 10^− 5^	0.042	1.710 × 10^− 6^	0.375	2.669 × 10^− 4^
**Henan**					
*r*_*s*_	0.771	0.766	0.728	0.655	0.759
*p*	2.652 × 10^− 21^	6.291 × 10^− 21^	4.647 × 10^− 18^	7.887 × 10^− 14^	2.288 × 10^− 20^
**Hong Kong**					
*r*_*s*_	− 0.094	− 0.514	− 0.282	0.517	− 0.085
*p*	0.349	3.394 × 10^− 8^	0.004	2.676 × 10^− 8^	0.398
**Hubei**					
*r*_*s*_	0.709	0.745	0.631	0.614	0.704
*p*	7.410 × 10^− 17^	2693 × 10^− 19^	1.131 × 10^− 12^	6.640 × 10^− 12^	1.640 × 10^− 16^
**Hunan**					
*r*_*s*_	0.813	0.797	0.738	−0.244	0.759
*p*	2.942 × 10^− 25^	1.256 × 10^− 23^	9.111 × 10^− 19^	0.014	2.300 × 10^− 20^
**Inner Mongolia**					
*r*_*s*_	0.322	0.129	0.369	0.385	0.316
*p*	0.001	0.197	1.384 × 10^− 4^	6.326 × 10^− 5^	0.001
**Jiangsu**					
*r*_*s*_	0.695	0.565	0.629	0.502	0.630
*p*	5.378 × 10^− 16^	5.918 × 10^− 10^	1.441 × 10^− 12^	7.609 × 10^− 8^	1.306 × 10^− 12^
**Jiangxi**					
*r*_*s*_	0.692	0.672	0.686	− 0.317	0.640
*p*	7.678 × 10^− 16^	1.052 × 10^− 14^	1.861 × 10^− 15^	0.001	4.433 × 10^− 13^
**Jilin**					
*r*_*s*_	0.538	0.446	0.626	0.323	0.355
*p*	5.415 × 10^− 9^	2.646 × 10^− 6^	1.925 × 10^− 12^	0.001	2.472 × 10^− 4^
**Liaoning**					
*r*_*s*_	0.575	0.425	0.486	− 0.221	0.513
*p*	2.685 × 10^− 10^	8.698 × 10^− 6^	2.179 × 10^− 7^	0.026	3.436 × 10^− 8^
**Macau**					
*r*_*s*_	0.105	0.016	0.093	0.204	0.015
*p*	0.293	0.872	0.354	0.040	0.882
**Ningxia**					
*r*_*s*_	0.696	0.649	0.541	−0.389	0.503
*p*	4.495 × 10^−16^	1.656 × 10^−13^	4.279 × 10^−9^	5.317 × 10^−5^	7.051 × 10^−8^
**Qinghai**					
*r*_*s*_	0.461	0.465	0.428	0.297	0.396
*p*	1.115 × 10^−6^	8.234 × 10^−7^	7.029 × 10^− 6^	0.002	3.833 × 10^−5^
**Shaanxi**					
*r*_*s*_	0.637	0.607	0.606	− 0.157	0.670
*p*	5.969 × 10^−13^	1.319 × 10^−11^	1.494 × 10^− 11^	0.115	1.406 × 10^−14^
**Shandong**					
*r*_*s*_	0.706	0.584	0.702	0.528	0.708
*p*	1.230 × 10^−16^	1.217 × 10^−10^	2.135 × 10^− 16^	5.238 × 10^−7^	9.317 × 10^−17^
**ShanghaiShanghai**					
*r*_*s*_	0.331	0.133	0.379	− 0.020	0.391
*p*	0.001	0.184	8.633 × 10^−5^	0.841	4.810 × 10^− 5^
**Shanxi**					
*r*_*s*_	0.380	0.275	0.313	0.001	0.365
*p*	8.102 × 10^−5^	0.005	0.001	0.991	2.382 × 10^−4^
**Sichuan**					
*r*_*s*_	0.775	0.687	0.720	0.681	0.771
*p*	1.247 × 10^−21^	1.565 × 10^−15^	1.588 × 10^−17^	3.530 × 10^− 15^	2.517 × 10^− 21^
**Tianjin**					
*r*_*s*_	0.483	0.424	0.517	0.295	0.453
*p*	2.675 × 10^−7^	9.050 × 10^−6^	2.624 × 10^−8^	0.003	1.755 × 10^− 6^
**Tibet**					
*r*_*s*_	0.167	0.139	0.173	−0.003	0.043
*p*	0.093	0.165	0.082	0.973	0.670
**Xinjiang**					
*r*_*s*_	0.737	0.704	0.593	−0.284	0.504
*p*	9.948 × 10^−19^	1.642 × 10^−16^	4.944 × 10^−11^	0.004	6.872 × 10^−8^
**Yunnan**					
*r*_*s*_	0.689	0.616	0.635	−0.340	0.638
*p*	1.274 × 10^−15^	5.308 × 10^−12^	7.636 × 10^−13^	4.776 × 10^−14^	5.252 × 10^−13^
**Zhejiang**					
*r*_*s*_	0.592	0.530	0.628	0.349	0.618
*p*	5.553 × 10^−11^	1.026 × 10^−8^	1.569 × 10^−12^	3.250 × 10^−4^	4.461 × 10^− 12^
**Taiwan**					
*r*_*s*_	−0.111	− 0.428	−0.242	0.523	−0.019
*p*	0.269	7.105 × 10^−6^	0.014	1.699 × 10^− 8^	0.854

### STCI analysis for people’s search behaviors of COVID-19-related symptoms and the epidemic of COVID-19

**Fig. 3:**
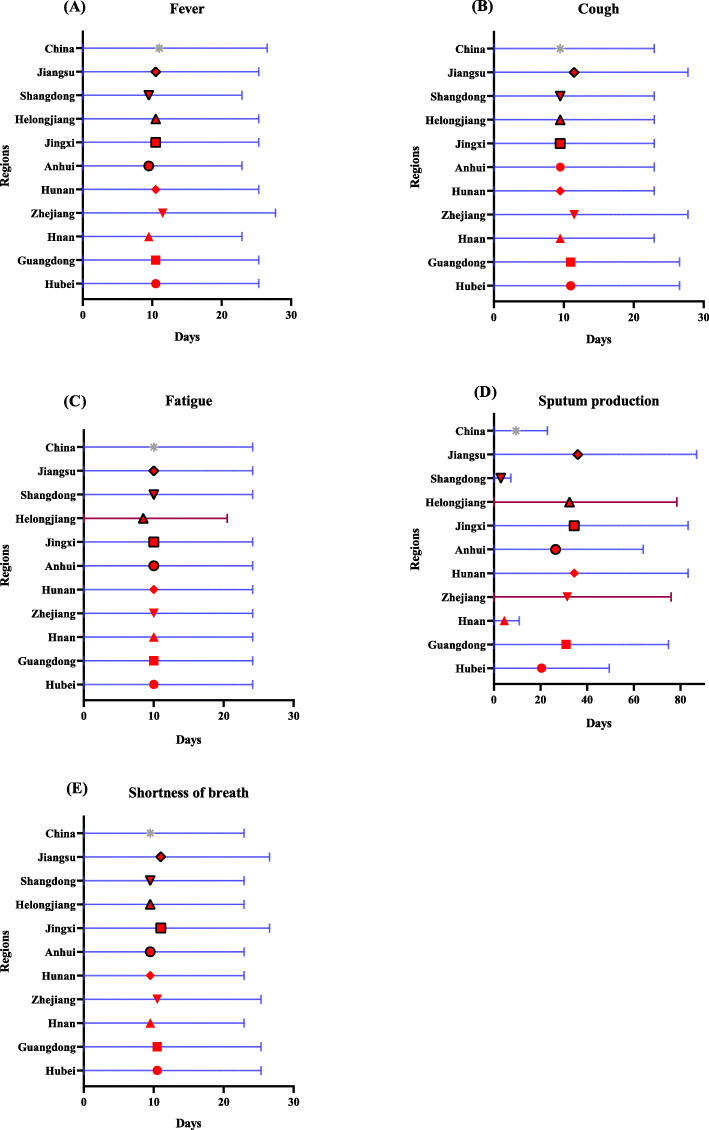
Days earlier of serarch-to-comfrimed interval (STCI) among the top ten provinces in the cumulative number of confirmed cases. e.g., The red line represents the absolute value of the negative value

Figure 3 shows that the peak of the growth rate of the Baidu Index occurred 19–22 days earlier than the peak of DGCC across china (STCI for fever: 22 days; cough: 19 days; fatigue: 20 days; sputum production: 19 days; shortness of breath: 19 days). Moreover, the top 10 provinces/regions ranked by confirmed cases presented similar results except for sputum production (Fig. 3). Oddly, the peak of the Baidu Index’s growth rate occurred 17 days later than the peak of DGCC in Heilongjiang.

### Lag correlation between the DGCC and search index values of COVID-19 related symptoms

**Fig. 4:**
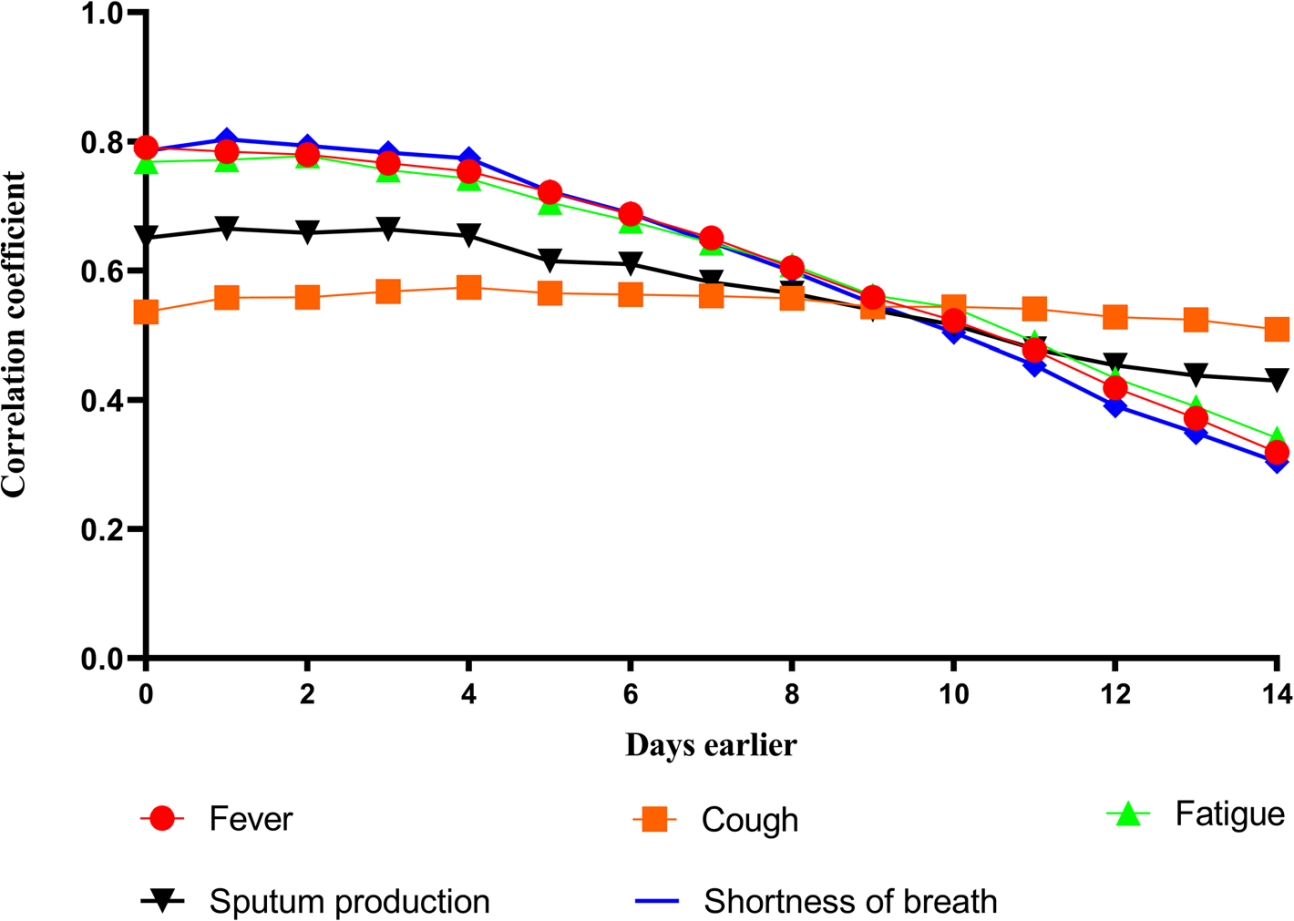
Lag correlations between the DGCC and each search topics of COVID-19-related symptom

Figure 4 and Table S2 manifests the lag correlation between DBIV of the different keywords and DGCC. We found the highest lag correlation with DBIV were 4 days earlier (*r*_*s*_ =0.574, *p*=1.826 × 10^− 10^) for cough, 2 days earlier *(r*_*s*_ =0.778, *p*=2.434 × 10^− 22^) for fatigue, 3 days earlier (*r*_*s*_ =0.664, *p*=1.630 × 10^− 14^) for sputum production, 1 day earlier (*r*_*s*_ =0.804, *p*=9.707 × 10^− 25^) for shortness of breath, and 0 days earlier for fever compared with the number of DGCC (*r*_*s*_ =0.791, *p*=1.623 × 10^− 23^).

## Discussion

People with the travel and exposure history of high-risk areas with COVID-19 patients will be required quarantined to control the spread of the pandemic. Since the understanding of the new coronavirus’s characteristics and the effective treatments remains uncertain, people usually compared COVID-19 with the SARS, which outbroke in 2003 in China with a mortality rate of 11% [19, 22]. Due to the separate isolation precautions policy and the fear of an unknown virus, people with exposure history are likely to conceal their own and their family’s high-risk behaviors, which undermines the government’s early attempts to control the suspected cases of COVID-19 [23]. Using Internet search engines, we could predict the potential quantity of affected persons; and the real-time data of the Baidu Index helps monitor the epidemic development and formulates the corresponding government policies.

China had achieved preliminary success in controlling the COVID-19 pandemic by April 22, 2020. The correlation analysis between Chinese public searches of COVID-19-related symptoms and the actual number of confirmed cases will be helpful for exploring the relationships between Internet search values and COVID-19 pandemic and provide novel insights for controlling the epidemic of COVID-19.

The current research shows that the related DBIV reached a peak earlier than the DGCC, and the dynamic changes of DBIV were also earlier than DGCC. We noticed that the higher the search values, the higher the cumulative confirmed cases will be during the growth period, which indicated that the searchers could be the potential infectors of the virus. Besides, DGCC and DBIV presented with a positive correlation during the whole observation period (even in the decline period), which implied the DBIV declined with a decreased number of DCGG. However, when DGCC was declining, the number of cumulative cases continued to increase instead, which could be an explanation for the negative correlation between cumulative cases and DBIV during the decline period. The public’s search behaviors for health-related information can reflect their potential physical and psychological problem [7, 24]. The declined searches of COVID-19-related symptoms indicated that the public’s mentality might be more relaxing in the decline period compared with the growth period.

We can tell from Baidu’s time plots for COVID-19-related symptoms and the number of confirmed cases that the former dynamic changes appeared earlier than the latter. Among 34 provinces/regions in China, although most areas in this research showed statistical correlations among the DBIV and DGCC (except sputum production), Hong Kong, Macao, Taiwan, and Tibet did not present with such correlations. One possible reason could be that the Baidu search engine is not the primary search tool in these places [4]. Additionally, there was only one confirmed case in Tibet, which was insufficient to conduct the statistical analysis. Besides, there was no correlation between DGCC and DBIV of cough in Shanghai, which might owe to the incompleteness of search keywords related to cough. Of interest, no correlation between DBIV of sputum production and DGCC was observed. A reasonable explanation could be that sputum production is more common in the elderly with chronic respiratory diseases, and such searches might be correlated to seasonal influenza every year in the late autumn to early spring [25]. Based on our research, the increase in the DBIV of COVID-19-related symptoms could be treated as an abnormal signal worthy of government departments’ corresponding action in advance.

The increased number of relevant searches indicates there are more potentially infected candidates. Around 97.5% of people with identifiable exposure history would develop symptoms within 11.5 days, and 1% of them had a more extended incubation period of more than 14 days [26]. We found that the average maximum of DBIV’s growth rate was 20 days earlier than DGCC in most areas except Heilongjiang. On May 10, 2020, the Heilongjiang government reported that the pandemic had relapsed; thus, the apex of DBIV appeared later compared with other provinces [27]. Compared with the traditional diagnosis and treatment process, most potential patients are inclined to search the Internet for help, indicating the difference to publicly reported overrepresent severe cases of COVID-19 [7, 28, 29]. Those potential infectors were likely to use search engines (usually Baidu in China) to search for the related information, so the Baidu index could reflect the approximate number of these potential infectors. The mild potential infectors may possess a more extended incubation period theoretically on account of several days lags before being confirmed [30]. The soaring DBIV of COVID-19-related symptoms in a certain area might be a precursor for the future outbreak of the epidemic. The STCI analysis shows that the peak DBIV of COVID-19-related symptoms appeared 19–22 days earlier than the peak DGCC. However, the results of the time-lag correlation analysis delivered a shorter lag than STCI. Since the STCI study only compared the interval between the peak DBIV of COVID-19-related symptoms and DGCC, it did not take other data into account. Therefore, time-lag correlation analysis could be better to explore the lag patterns of DBIV and DGCC. We found that the optimal time lag of DBIV of fever, cough, fatigue, sputum production, and shortness of breath was 0, 4, 2, 3, 1 day/days, respectively. According to Cuilian et al., the peak of Internet searches about COVID-19 appeared 10–14 days earlier than the peak of reported daily growth cases in China [31], and 10 days earlier in America [32]. People who searched the terms of “新冠” or “冠状病毒” (keywords in Cuilian’s study) were more likely to experience the incubation period, while the searchers querying the COVID-19-related symptoms were likely those who were infected and had already experienced the incubation period. Moreover, there is no time-lag for “fever”; this may attribute to the body temperature reporting mechanism adopted by both the Chinese government and local institutions. This reporting system required that people with fever be actively isolated and quarantined immediately to prevent the potential further spread of COVID-19 [33, 34]. Therefore, people with fever would be isolated and confirmed subsequently. As a result, no time lag was observed.

### Limitations

There are some limitations needed to be recognized. Firstly, we only utilized Baidu’s data to perform our research; other search engines, such as Weibo and Twitter, were not included. Secondly, some keywords related to the symptoms of COVID-19 were not included in the current study, and the keywords utilized in the current work could not guanine the consistency and efficiency of the long-term prediction in the future. Therefore, future studies are suggested to add or delete the corresponding keywords of COVID-19-related symptoms to confirm that the time lag patterns exist between DBIV and DGCC. Thirdly, the detailed information about the individual searchers remains unclear, so it is impossible to identify the specific potential infectors. Besides, there were several documented issues with the predictability of disease incidence trends using search engines. To avoid the failure of predicting an epidemic with the utilization of the Internet search engine, a random forest regression model is suggested in the future study to facilitate our observing results [35].

## Conclusion

Our research suggested that there was a significant correlation between DBIV of COVID-19-related symptoms and DGCC. The dynamic changes of DGCC showed several days lags compared with the DBIV. Besides, DBIV of COVID-19-related symptoms could serve as a potential indicator for predicting the epidemic of emerging infectious diseases and guide targetable intervention and prevention of COVID-19 to further assist in the overall control of the pandemic.

## Supplementary Information

**Additional file 1: Table S1.** Search terms for topics regarding top five symptoms of COVID-19 in Chinese.

**Additional file 2: Figure S1.** Search trend of keywords related to COVID-19 symptoms among 2011~2020.

**Additional file 3: Figure S2.** Correlation and time plots among cumulative confirmed cases and each keyword of COVID-19-related symptoms during the growth period. (A), (B), (C), (D), and (E) represent Baidu searches for “fever”, “cough”, “fatigue”, “sputum production”, and “shortness of breath”, respectively.

**Additional file 4: Figure S2.** Correlation and time plots among cumulative confirmed cases and each keyword of COVID-19-related symptoms during the decline period. (A), (B), (C), (D), and (E) represent Baidu searches for “fever”, “cough”, “fatigue”, “sputum production”, and “shortness of breath”, respectively.

**Additional file 5: Table S2.** Lag correlation coefficients and *p* values between search index values of each keyword and daily confirmed cases.

## Data Availability

The data that support the findings of this study are available from the corresponding author upon reasonable request.

## Abbreviations

CSSE: Center for Systems Science and Engineering
JHU: Johns Hopkins University
COVID-19: New coronavirus disease 2019
STCI: Search-to-confirmed interval
DBIV: Daily Baidu Index values
DGCC: Daily growth of confirmed cases
WHO: World Health Organization
