# Supplementary material for: Using Baidu search values to monitor and predict the confirmed cases of COVID-19 in China: – evidence from Baidu index
Source: BMC Infect Dis. 2021 Jan 21;21:98. doi: 10.1186/s12879-020-05740-x (PMC7819631; doi:10.1186/s12879-020-05740-x)
Supplement: Supplementary file 1 — Additional file 1: Table S1. Search terms for topics regarding top five symptoms of COVID-19 in Chinese. [file 12879_2020_5740_MOESM1_ESM.docx]

| **Table S1.**  Search terms for topics regarding top five symptoms of COVID-19 in Chinese | | | | |
| --- | --- | --- | --- | --- |
| Search topic |  | Key words of each topic |  | Number of the key words |
| Cough |  | 咳嗽+干咳+咳嗽怎么治最有效+咳嗽有痰吃什么好的快+止咳最快的偏方 |  | 5 |
| Fever |  | 发热+腋下37.2度算发烧么+正常体温+体温正常值+多少度算发烧+低烧+发烧+腋下体温多少度算正常 |  | 8 |
| Fatigue |  | 乏力+乏力是什么意思 |  | 2 |
| Sputum production |  | 痰多+痰+咳痰+止咳化痰+ 去痰最好最快的方法 |  | 5 |
| Shortness of breath |  | 气促+胸闷+呼吸困难+胸闷气短+憋气+喘息 |  | 6 |
