# Supplementary figures and images for: Using Baidu search values to monitor and predict the confirmed cases of COVID-19 in China: – evidence from Baidu index
Source: BMC Infect Dis. 2021 Jan 21;21:98. doi: 10.1186/s12879-020-05740-x (PMC7819631; doi:10.1186/s12879-020-05740-x)

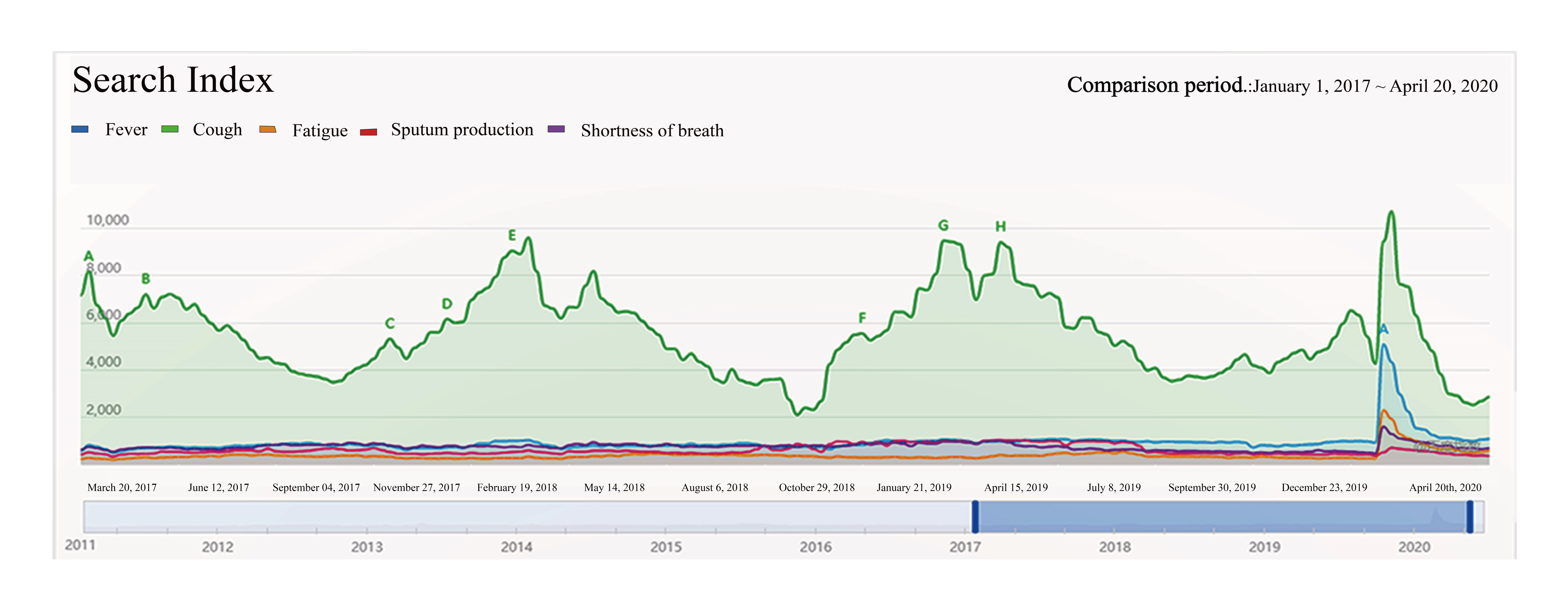

Supplement: Supplementary file 2 — Additional file 2: Figure S1. Search trend of keywords related to COVID-19 symptoms among 2011~2020. [file 12879_2020_5740_MOESM2_ESM.tif]
