# Supplementary material for: Using Baidu search values to monitor and predict the confirmed cases of COVID-19 in China: – evidence from Baidu index
Source: BMC Infect Dis. 2021 Jan 21;21:98. doi: 10.1186/s12879-020-05740-x (PMC7819631; doi:10.1186/s12879-020-05740-x)
Supplement: Supplementary file 5 — Additional file 5: Table S2. Lag correlation coefficients and p values between search index values of each keyword and daily confirmed cases. [file 12879_2020_5740_MOESM5_ESM.docx]

Table S2. Lag correlation coefficients and *p* values between search index values of each keyword and daily confirmed cases

| Lag times （days） | Keywords | | | | | | | | | |
| --- | --- | --- | --- | --- | --- | --- | --- | --- | --- | --- |
|  | Fever | | Cough | | Fatigue | | Sputum production | | Shortness of breath | |
|  | *r_s_* | *p* | *r_s_* | *p* | *r_s_* | *p* | *r_s_* | *p* | *r_s_* | *p* |
| 0 | 0.791 | 1.623x10^-23^ | 0.536 | 4.579x10^-9^ | 0.769 | 1.649x10^-21^ | 0.651 | 7.379x10^-14^ | 0.786 | 5.376x10^-23^ |
| 1 | 0.785 | 5.968x10^-23^ | 0.558 | 7.643x10^-10^ | 0.772 | 9.149x10^-22^ | 0.664 | 1.336x10^-14^ | 0.804 | 9.707x10^-25^ |
| 2 | 0.780 | 1.624x10^-22^ | 0.559 | 7.124x10^-10^ | 0.778 | 2.434x10^-22^ | 0.659 | 2.690x10^-14^ | 0.794 | 7.950x10^-24^ |
| 3 | 0.767 | 2.434x10^-21^ | 0.568 | 3.205x10^-10^ | 0.756 | 1.600x10^-20^ | 0.664 | 1.630x10^-14^ | 0.783 | 9.623x10^-23^ |
| 4 | 0.754 | 2.649x10^-20^ | 0.574 | 1.826x10^-10^ | 0.743 | 1.652x10^-19^ | 0.654 | 5.099x10^-14^ | 0.774 | 5.857x10^-22^ |
| 5 | 0.722 | 5.486x10^-18^ | 0.565 | 4.184x10^-10^ | 0.706 | 5.744x10^-17^ | 0.615 | 3.679x10^-12^ | 0.723 | 4.422x10^-18^ |
| 6 | 0.688 | 7.472x10^-16^ | 0.563 | 4.974x10^-10^ | 0.677 | 3.190x10^-15^ | 0.610 | 6.540x10^-12^ | 0.689 | 6.305x10^-16^ |
| 7 | 0.651 | 7.089x10^-14^ | 0.561 | 5.694x10^-10^ | 0.643 | 1.869x10^-13^ | 0.582 | 9.590x10^-11^ | 0.644 | 1.627x10^-13^ |
| 8 | 0.605 | 1.021x10-^11^ | 0.557 | 8.261x10^-10^ | 0.609 | 6.754x10^-12^ | 0.565 | 4.211x10^-10^ | 0.599 | 1.879x10^-11^ |
| 9 | 0.559 | 7.206x10^-10^ | 0.544 | 2.361x10^-9^ | 0.562 | 5.492x10^-10^ | 0.539 | 3.474x10^-9^ | 0.549 | 1.588x10^-9^ |
| 10 | 0.523 | 1.276x10^-8^ | 0.544 | 2.993x10^-9^ | 0.543 | 5.198x10^-9^ | 0.516 | 2.040x10^-8^ | 0.504 | 5.013x10^-8^ |
| 11 | 0.477 | 3.037x10^-7^ | 0.541 | 8.121x10^-9^ | 0.490 | 1.312x10^-7^ | 0.479 | 2.667x10^-7^ | 0.454 | 1.319x10^-6^ |
| 12 | 0.419 | 9.458x10^-6^ | 0.528 | 1.140x10-^8^ | 0.434 | 4.264x10^-6^ | 0.454 | 1.294x10^-6^ | 0.391 | 4.088x10^-5^ |
| 13 | 0.372 | 1.030x10^-4^ | 0.524 | 1.631x10^-8^ | 0.390 | 4.292x10^-5^ | 0.438 | 3.043x10^-6^ | 0.349 | 2.773x10^-4^ |
| 14 | 0.319 | 0.001 | 0.509 | 2.850x10^-8^ | 0.341 | 3.951x10^-4^ | 0.430 | 5.339x10^-6^ | 0.304 | 0.002 |
